# Tracing ΦX174 bacteriophage spreading during aerosol-generating procedures in a dental clinic

**DOI:** 10.1007/s00784-023-04937-z

**Published:** 2023-03-18

**Authors:** Edgar O. Beltrán, Jaime E. Castellanos, Zayda L. Corredor, Wendy Morgado, Olga L. Zarta, Andrea Cortés, Viviana Avila, Stefania Martignon

**Affiliations:** 1 UNICA - Caries Research Unit, Research Department, Universidad El Bosque, Av. Cra 9 No. 131A-02, 110121 Bogotá, Colombia; 2 Laboratorio de Virología, Vicerrectoría de Investigaciones, Universidad El Bosque, Av. Cra 9 No. 131A-02, 110121 Bogotá, Colombia; 3 Grupo de Investigaciones Básicas y Aplicadas en Odontología, Universidad Nacional de Colombia, Carrera 30 No. 45-03, Edificio 210, 111321 Bogotá, Colombia; 4 Bacterial Molecular Genetics Laboratory, Research Department, Universidad El Bosque, Cra 9 No. 131A-02, 110121 Bogotá, Colombia; 5 Department of Exact and Natural Sciences, Universidad de la Costa, Calle 58 No. 55-66, 080002 Barranquilla, Colombia; 6 Dental School, Universidad El Bosque, Cra 9 No. 131A-02, 110121 Bogotá, Colombia

**Keywords:** Bacteriophage, Aerosol, Aerosol-generating procedure, Dental setting, Virus laden aerosols, Airborne transmission

## Abstract

**Objective:**

The aim of this study was to test the plausibility of using the ΦX174 bacteriophage as a tracer of viral aerosols spreading in a dental aerosol-generating procedure (AGP) model.

**Methods:**

ΦX174 bacteriophage (~ 10^8^ plaque-forming units (PFU)/mL) was added into instrument irrigation reservoirs and aerosolized during class-IV cavity preparations followed by composite fillings on natural upper-anterior teeth (*n* = 3) in a phantom head. Droplets/aerosols were sampled through a passive approach that consisted of *Escherichia coli* strain C600 cultures immersed in a LB top agar layer in Petri dishes (PDs) in a double-layer technique. In addition, an active approach consisted of *E coli* C600 on PDs sets mounted in a six-stage cascade Andersen impactor (AI) (simulating human inhalation). The AI was located at 30 cm from the mannequin during AGP and afterwards at 1.5 m. After collection PDs were incubated overnight (18 h at 37 °C) and bacterial lysis was quantified.

**Results:**

The passive approach disclosed PFUs mainly concentrated over the dental practitioner, on the mannequin’s chest and shoulder and up to 90 cm apart, facing the opposite side of the AGP’s source (around the spittoon). The maximum aerosol spreading distance was 1.5 m in front of the mannequin’s mouth. The active approach disclosed collection of PFUs corresponding to stages (and aerodynamic diameters) 5 (1.1–2.1 µm) and 6 (0.65–1.1 µm), mimicking access to the lower respiratory airways.

**Conclusion:**

The ΦX174 bacteriophage can be used as a traceable viral surrogate in simulated studies contributing to understand dental bioaerosol’s behavior, its spreading, and its potential threat for upper and lower respiratory tract.

**Clinical relevance:**

The probability to find infectious virus during AGPs is high. This suggests the need to continue characterizing the spreading viral agents in different clinical settings through combination of passive and active approaches. In addition, subsequent identification and implementation of virus-related mitigation strategies is relevant to avoid occupational virus infections.

## Introduction

Control of infections in indoor spaces has gained special attention in the context of the severe acute respiratory syndrome coronavirus 2 (SARS-CoV-2) [1–4]. Dental settings correspond to indoor spaces where saliva splatter and droplets are generated physiologically and through aerosol-generating procedures (AGPs) during operative/invasive procedures [5]. Large droplets and splatter (diameter > 50 μm) tend to fall ballistically to the ground close to the source (1–2 m). In contrast, small droplets (diameter ≤ 50 μm) tend to evaporate into droplet nuclei (diameter < 10 μm), remaining suspended in the air for large periods of time or can still travel large distances as a cloud [6]. Droplets and aerosol particles generated during dental care might be contaminated with bacteria, viruses, and fungi, forming infectious bioaerosols [7, 8].

Aerosolization in dental settings has been assessed through splatter/droplets/aerosols generation using fluorescent tracer markers [9–12], and collection methods such as passive culture methods onto culture plates [13–15] and active air sampling [16, 17]. Despite existing evidence, the identification of virus-laden aerosols in dental practice has not been studied in depth.

The Andersen six-stage cascade impactor sampler is an active air sampler that simulates human inhalation by collecting particles in six stages according to their aerodynamic diameter: > 7.0 μm, 4.7–7.0 μm, 3.3–4.7 μm, 2.1–3.3 μm, 1.1–2.1 μm, and 0.65–1.1 μm. At a constant flow rate of 28.3 L/min, the AI mimics the entrance and circulating of particles that based on their aerodynamic size penetrate the nasal cavity, pharynx, trachea and primary bronchi, secondary bronchi, terminal bronchi, and alveoli, respectively [18].

Bacteriophage have been widely used as viral airborne surrogates in aerovirology due to their harmlessness [19]. In addition, bacteriophages can be found with a high diversity of genetic and morphological properties [20]. Some display structural phenotypic and genotypic features similar to eukaryotic viruses [20]. In aerosol studies, several bacteriophages have been proposed as pathogenic viral surrogates, including MS2 (a single-stranded RNA (ssRNA) bacteriophage of the *Leviviridae* family); Φ6 (a segmented double-stranded RNA (dsRNA) bacteriophage of the *Cystoviridae* family), and ΦX174 (a single-stranded DNA (ssDNA) bacteriophage of the *Microviridae* family) [21]. The ΦX174 bacteriophage is a non-enveloped bacteriophage, with a 5386 nt linear ssDNA and 25 nm in diameter that uses *E. coli* species as host [20, 22, 23].

The aim of this study was to test the plausibility of using the ΦX174 bacteriophage as a traceable surrogate of viral aerosols spreading during a dental AGP model.

## Materials and methods

Ethical approval was obtained from the Institutional Ethics Committee of the Universidad El Bosque (UEB-561). The Department of Biological Sciences of Universidad de Los Andes (Bogotá, Colombia) provided the bacteria and bacteriophages used in this study. The ΦX174 bacteriophage was grown on *Escherichia coli* strain C600 (*E coli* C600) (Migula) Castellani and Chalmers (ATCC 23,724), which is a bacteria-sensitive strain to ΦX174 infection [24]. Bacteriophage and bacterial host cells were incubated in 10 mL of Luria–Bertani (LB) nutrient broth for 18 h at 25 °C, 150 rpm (Forma Scientific). The bacteriophage lysate was titrated on the respective bacterial host using standard double-layer technique (Difco Laboratories, Detroit, MI) (0.4%) [24, 25].

### Aerosolization conditions

**Fig. 1:**
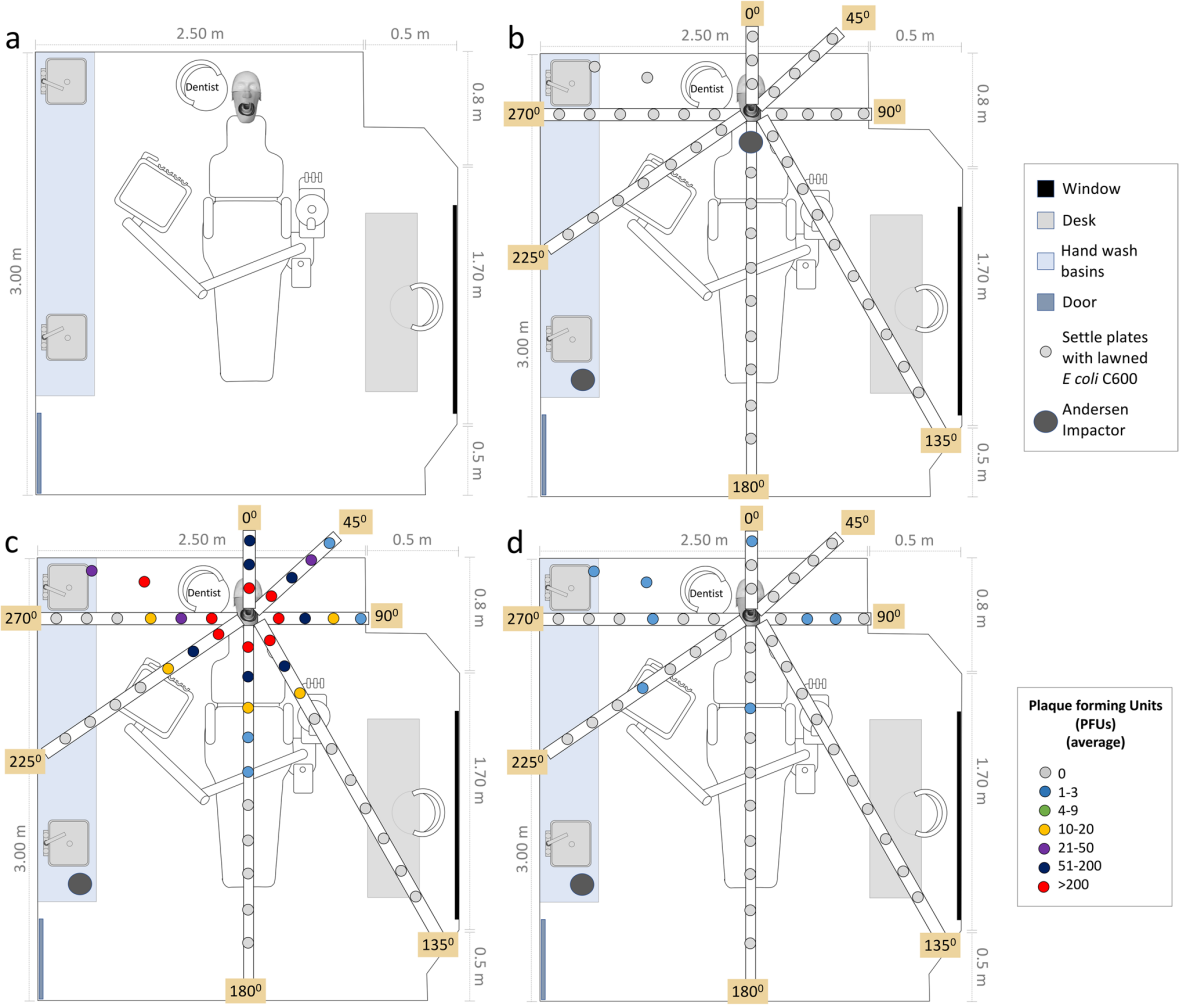
Dental setting setup and droplets/aerosol collection positions: **a** Schematic diagram of experimental dental office setup. Mannequin and DP position in relation with the window and door. **b** Position of passive and active collection of ΦX174 bacteriophage-laden droplets/aerosols (Note: degrees are relative to phantom head and mouth positions). **c** Schematic representation of the average of ΦX174 bacteriophage PFUs detected in each position in T1. Colors represent differences in a randomized score of PFUs. The Andersen impactor’s position in T1 was 30 cm in front of the mannequin mouth, and around 200 grades opposite to this position when activated 1 hour after the procedure (T4). **d** Schematic reduction of ΦX174 bacteriophage PFUs after 40–55 min post-AGP (T3)

Experiments were conducted by triplicate in an adapted single-unit dental office (3 × 3 × 2.5 m) (Fig. 1a) without controlled ventilation and with or without low-volume suction (LVS). Temperature, humidity, and barometric pressure were measured using an anemometer Kestrel 4500–710,830. A dental phantom head (Nissin Dental Products) was adapted to fit in the dental chair. Upper anterior natural teeth (*n* = 6) were collected from the teeth bank in Universidad El Bosque and stored in 0.2% of thymol-diluted deionized water at 4 °C (ethical approval: 012–2017). Class-IV cavity preparations and fillings were conducted always by the same trained practitioner with (*n* = 3) and without LVS (*n* = 3). SM buffer (10 mM Tris–HCl, 100 mM NaCl, 10 mM MgSO_4_, adjusted to pH 7.4) containing ΦX174 bacteriophage (~ 10^8^ plaque-forming units (PFU)/mL) was introduced in the unit water tank that feeds the high-speed handpiece and the 3-in-1 syringe. During aerosolization, an assistant provided the materials needed for each procedure. A KaVo high-speed air turbine handpiece (KaVo Dental GmbH; 200,000 rpm; water flow rate 22 mL/min, air pressure 36 psi) was used with diamond burs (Hidi-Once Diamond Bur Med; Dentsply). A low-speed handpiece (NSK S-Max M95L electric; 60,000 rpm; water flow rate 60 mL/min) was used to polish the fillings. Each procedure was performed three times by the same trained practitioner. Previous on-site meetings were conducted to discuss the flowchart of the procedure and the time of each step (e.g., aerosolization, cavity preparation, filling). New aseptic personal protective equipment (PPE) was donned for each procedure to prevent cross-contamination between experiments. Only the dental practitioner (DP) and an assistant were in the dental office during AGPs. Post-procedure, the assistant (wearing a new PPE each time) sealed settle plates and replaced them to avoid cross-contamination measures. The AGP consisted of 6 min of active handpiece use followed by the complete filling procedure (5 min) and a final polishing (2 min), for a total time of 13 min.

### Bacteriophage aerosol detection

Passive and active sampling methods were used to monitor the spreading of viral aerosols through the collection of aerosols on settlement plates in a standard double-layer technique. In both methods, *E coli* C600 [OD600 0.6] was used to detect infectious aerosols and droplets produced during the simulated cavity-preparation and dental fillings in triplicate. The agar double layer consisted of a bottom layer with 1.2% agar and a top layer with 0.7% agar [21]. In the passive approach, PDs were positioned around the mannequin on seven bands located at different angles and behind the operator, from 30 cm and up to 2 m from the mannequin’s mouth depending on the available space in a clockwise direction (*n* = 44), during AGP and post-AGP in the clinical environment (Fig. 1b). PDs were collected and replaced at the different time points, except for the last set.

Settle plates were exposed during the 10-min pre-AGP to determine previous bacteriophage contamination or bioaerosol carryover from the previous experiment in each situation (background control, T0). After conducting the whole procedure, an additional time of 15 min was considered as the fallow time to collect the first set of PDs (T1). Immediately after, new sets of *E. coli* cultures were positioned around the mannequin (Fig. 1b) and replaced from 20 to 180 min as follows: T2 (20–35 min), T3 (40–55 min), T4 (60–75 min), T5 (80–95 min), T6 (100–115 min), T7 (120–150 min), and T8 (155–180 min).

PDs were positioned on the operator’s right shoulder, chest, face shield, and N95 mask (one each; *n* = 4) as well as on the mannequin’s chest (*n* = 1), shoulders (*n* = 2), and face (*n* = 2). These PDs were collected at T1.

Furthermore, an active collection was conducted with PDs mounted in an Andersen impactor (AI) air sampling device (Thermo Fisher Scientific, Waltman, MA, USA). In this approach, six 11-cm-diameter PDs with *E coli* C600 immersed in a LB top agar layer were used. This device was set 30 cm in front of the mannequin oral cavity and operated to sample 28.4 L/min up to 20 min immediately after starting the AGP. Afterwards, at the beginning of T4 (60 min), the Andersen impactor was cleaned, disinfected, and mounted again to operate at 1.5 m away and around 200° from the AGP source for 20 min (Fig. 1b). Culture plates collected from each aerosolization were incubated at 37 °C overnight (18 h). At this time, a solution of 0.1% Naphthol blue/black (Cat. 3393; Sigma) was used to maximize the detection of PFUs. PFU counts were conducted in consensus by trained researchers (EOB and ZLC), as previously reported [25].

### Data analysis

All analyses were performed using StataVR10.0 statistical software (StataSE Corp LP, College Station, TX, USA). Shapiro–Wilk test was applied to assess the distribution of the obtained data. Two-way ANOVA was used to examine differences between the viral aerosol collected in each position and time.

## Results

### Practitioner and mannequin exposure to viral aerosols

Environmental conditions during the experiments presented ranges of temperature and humidity of 16–19 °C and 77–85%, respectively. No PFUs were found in the background controls set before the AGPs. Data corresponding to the conduction of the study without LVS (control) can be found in the Appendix. Results from the experiments using LVS and corresponding to the real scenario in the clinical practice will be described ahead.

**Fig. 2:**
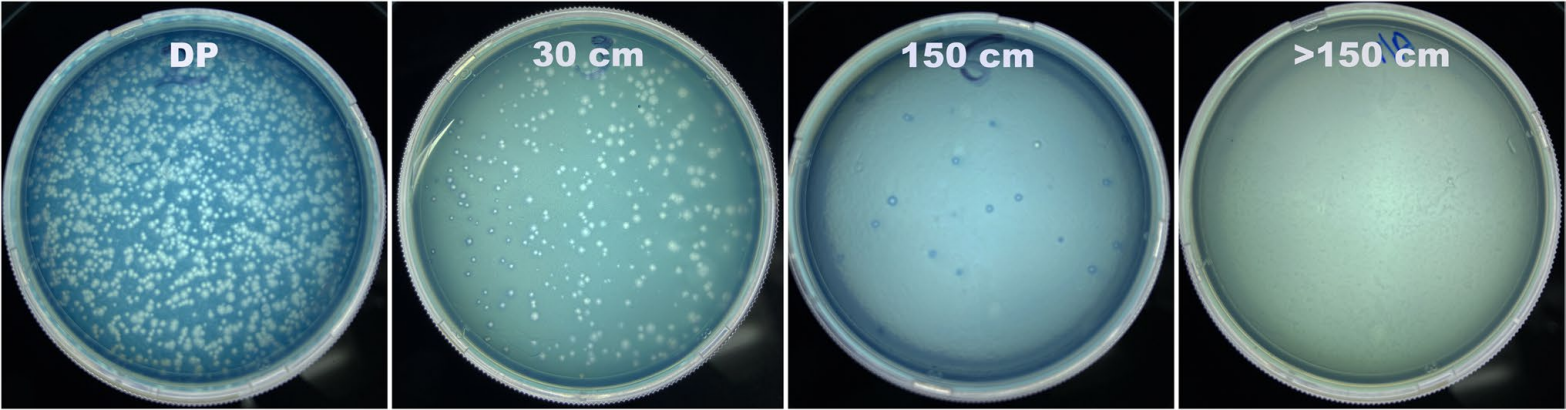
Examples of bacteriophage collection in T1 at different positions and distances from the DP (dental practitioner). Each “*space*” in the *E. coli* layer indicates a PFU where the infection of the ΦX174 bacterial host took place

In T1, high amounts of PFUs were found on the *E coli* C600 cultures placed over the mannequin (head front: 1243.3 ± 11.6 PFUs; chest: 1275.3 ± 11.0 PFUs; right shoulder: 691.6 ± 15.9), on the dental practitioner (head front: 3.3 ± 3.0 PFUs; chest: 983.3 ± 18.1; over the face shield: 1114.6 ± 50.12 and under the face shield: 14.3 ± 2.0 PFUs) and those located closer to the operator and 30–60 cm from the AGP source next to the mannequin. A statistically significant difference was found between the PFUs in these positions versus all the other assessed locations (*p* < 0.005). ΦX174 bacteriophage PFUs were detected on PDs placed on the operator (100%) even under the face shield when the teeth were drilled. Different size and morphology of PFUs were found in the different positions assessed. A representative image is found in Fig. 2.

### Generation and persistence of viral aerosols in the air

In the passive approach, in T1, AGPs on the upper anterior teeth generated on average a maximum of 1586 ± 128.6 PFUs at 30 cm. In this same position, in T2, infectious viral particles able to infect *E coli* C600 decreased in number (maximum 19 ± 16.6 PFUs) and became undetectable in T4. In contrast, in T3 PFUs were detected at 60, 90, 120, and up to 180 cm from the AGP source (Table 1; Fig. 1c, d). In the positions of 60, 90, and 120 cm from the mannequin mouth, a decreasing mean number of PFUs was found in comparison to that in the position corresponding to 30 cm, as the fallow time passed (T1 vs. T2, T3, and T4) (*p* < 0.05). In further positions and times assessed, no PFUs were detected. In the passive approach, the maximum distance traveled by the viral aerosols corresponded to 150 cm.

**Table 1:**
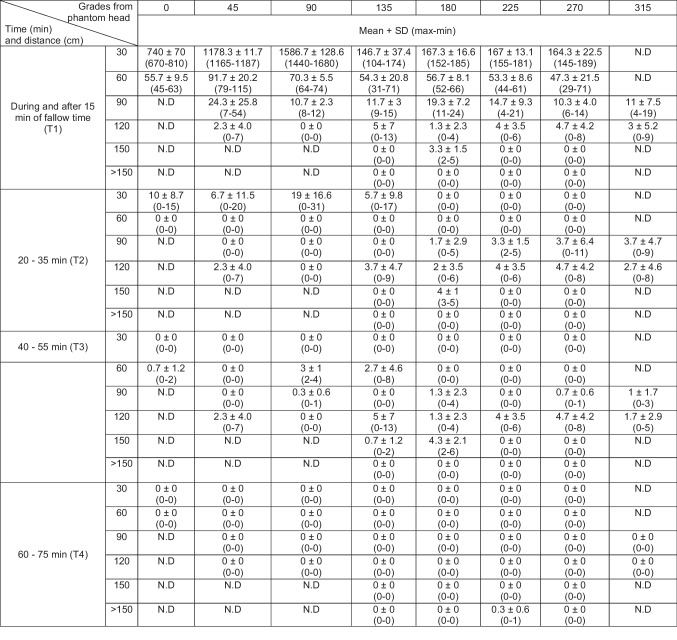
Mean number of PFU counts at different grades and distance in each time point from T1 to T4

Times not shown indicate an absence of PFUs in the assessment. Average, standard deviations, and maximum/minimum PFU counts are shown

*ND*: no data collected at the indicated positions

**Fig. 3:**
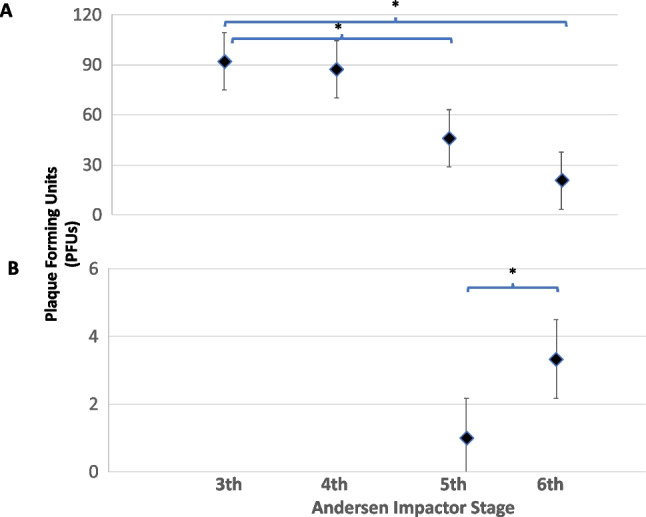
Active sampling of ΦX174 bacteriophage-laden droplets and aerosols. **A** In T1, clear PFUs were observed from the 3rd stage of the Andersen impactor. The number of PFUs decreased until the 6th stage (*p* < 0.05). **B** In T4, PFUs were found in the 5th and 6th stages (distance = 2 m), corresponding to 1.1–2.1 µm and 0.6–1.1 µm (bronchioles and alveoli compartments in the lower respiratory tract), respectively. *Statistically significant differences

In the active approach using the AI, in T1 at 30 cm from the mannequin mouth, the large production of droplets and its virus load led to a vast disruption in the bacteria growing in the first and second stages of the Andersen impactor. As a result, no plaques can be observed. In contrast, on average a maximum of 92.08 ± 50.5 PFUs were found in the third stage, and decreasing number of PFUs were detected in the lower stages (*p* < 0.05). In T4, we only found PFUs in the fifth and sixth stages in the Andersen impactor (distance = 1.5 m). These stages correspond to 1.1–2.1 µm (fifth stage) and to 0.6–1.1 µm (sixth stage) (Fig. 3A, B).

## Discussion

In this work, we found plausible the use of the ΦX174, a non-enveloped bacteriophage, as a viral surrogate to trace viral aerosols’ spreading in a dental AGP model. As large amounts of viral-laden splatter, microdroplets, and aerosols were produced in the present model, the findings of this study could contribute to understand the viral spreading in the dental setting combining a settlement-plate technique and an active approach (Andersen impactor). This assessment showed that both techniques allowed the viral recovery in a distance and time dependent manner.

To our knowledge, this is the first study successfully reporting the use of the ΦX174 bacteriophage as a tracer in dental AGPs. This contributed to validate the usefulness of the model when natural teeth are being drilled, allowing the development of future assessments related to aerosol mitigation strategies based on it. The evidence regarding this type of studies is scarce; only two other research groups have explored this aspect in depth by using other surrogate viruses, namely, MS2 [5] and Φ6 [26]. Those studies inoculated salivary bacteriophage directly to the mouth across two or three positions to recreate the salivary gland secretion. In contrast with their work, here the ΦX174 bacteriophage was diluted in SM buffer and added to the instrument-irrigation reservoir to generate viral aerosols during the procedures. The findings of both approaches highlight the detection of large amounts of viral load in droplets/aerosols.

The rationale for using the ΦX174 bacteriophage relies on several aspects, including it being well characterized, safe, and relatively easy to work with; it was previously used as a surrogate for pathogenic virus [22, 23, 27, 28].

With regards to the concentration of bacteriophage, in this study an inoculum of ~ 108 PFU/mL ΦX174 bacteriophage was used to mimic the viral load found in saliva samples from infected individuals, as reported previously [26]. This concentration represents the worst-case scenario based on differential viral load in saliva that depends on the nature of the viruses [29–34].

The settlement-plate collection in this study showed a high amount of PFUs close to the AGPs source, in accordance with previous studies that reported similar findings [6]. A possible explanation for this could be the fact of that large droplets and splatter tend to fall ballistically to the ground close to the source (1–2 m). In contrast, the amount of PFUs detected here with the Andersen impactor was low. It should be related to the greater distance between this device and the AGP source. In that regard, the impactor was recently used to characterize viable virus particles of SARS-CoV-2, finding it mostly in an aerodynamic diameter of 3.3 µm [35]. The relevance of the detection of viruses in bioaerosols relies on the feasibility to penetrate the lungs and become embedded in alveoli depending on the aerodynamic size and the concentration of the particle [36–38].

Interestingly, in the current study PFUs were found in different size and morphology. It could be associated with either the bacteriophage travel through different compartments before being collected, the sampling techniques, or with differential viral load coming from varying aerodynamic diameter droplets/aerosols [24]. Because of its structure, the ΦX174 bacteriophage has widely used as a surrogate for pathogenic ssDNA viruses [21, 23]; however, indoor bioaerosols vary in size and composition, and many factors may influence their dispersion and survival in the indoor environment. One single bioaerosol particle may be composed of fine or coarse particle matter, ion organic structures, microorganisms, and allergen proteins, which results in particles of different aerodynamic sizes independent of the size of their components (bacteria, fungi, or viruses) [36]. In addition to the PFU detection, other tests could be implemented as real-time polymerase chain reaction to complement the identification of the ΦX174 genome [5], even when PFUs are not observed.

A surprising finding was that virus PFUs were found in the study even under the practitioner’s face shield. This suggests the potential risk of the aerosolized particles generated during AGPs and their ability to cross biosafety barriers as the face shield [3, 39]. For this reason, different international statements have emphasized the need to strengthen biosafety measurements due to the feasibility of airborne virus transmission through droplets and aerosols from AGPs [3, 40–42]. Similar results have been shown in a model of airborne bacteria and endotoxin aerosolized in the frame of dental cleaning [17]. In addition, this finding has also been recently reported in a prospective, randomized clinical trial, in which differential concentrations of *Streptococcus* spp., *Staphylococcus* spp., *Micrococcus* spp., and *Bacillus* spp. were found in the face mask after conducting single-tooth fillings or periodontal supportive treatments [43]. Altogether, these studies show that face shields lack conferring complete protection against bioaerosol, although this has been proposed as one of the key PPE to avoid infections [3, 40–42]. Additional studies to improve the protection against bioaerosols should be conducted using masks, goggles, and face shield, independently and combined.

In this study, the use of LVS did not reduce significantly ΦX174-laden droplets and aerosols. Studies have reported successful aerosol control using high-volume suction (HVS). For instance, Vernon et al. combined the high-speed contra-angle handpiece with HVS during AGPs detecting no viable Φ6 bacteriophage post-procedure [26]. In addition, HVS together with rubber dam has shown significant reduction in ultrafine dental aerosol particles and in the concentration of total particulate matter. However, this last study did not use bacteriophage as a tracer [44].

This is the first report in Latin America using bacteriophages in a dental clinic simulated scenario pointing out the high amounts of viral aerosols spreading on the practitioner, the patient, and in the breathing zone. In Colombia and in most countries of the region, different weather conditions are present because of their location (many in the Equator line and thus tropical) and dental settings do not have controlled ventilation, as the aerosol behavior depends on the temperature, geographical location, and altitude. Regarding this fact, it has been suggested that aerosol concentration could be significant in dental offices with poor ventilation [3, 45–52]. In contrast, most related experimental procedures currently conducted use environments with mechanical ventilation. This situation and the lack of evidence about how to manage aerosols in different dental settings highlight an emerging additional problem and contribute to the increase of stress in the dental practice [53].

Future research could focus on strategies to mitigate ΦX174 aerosols including HVS and engineering controls (e.g., air cleaners). Besides, the patient breathing simulation or any other behavior associated with the bioaerosol generation (talking, coughing) as well as the determination of the period of fallow time might be explored. Special strategies should be implemented regarding their mitigation [37–39].

## Conclusion

The ΦX174 bacteriophage can be used as a traceable viral surrogate in studies aiming to understand dental bioaerosol’s behavior, its spreading, the potential threat of virus-laden aerosols for upper and lower respiratory tract, and further mitigation strategies.

## Data Availability

Data will be available upon request.

## Appendix

PFU counts from T1 to T4 in absence of LVS
